# 

**Published:** 1916-12-23

**Authors:** 


### Hospital and Institutional Results in 1915.

**The South American Missionary Society.**—Fearing that the special funds before the public in connection with the war would have a reactionary effect upon the finance of missionary work, the Society has been content to "mark time" in respect of its operations. Subscriptions and donations have fallen off, but there was an increase in the amount of legacies paid during the year ending March 1916. Three medical missionaries have taken up military service.

**West Riding Pauper Lunatic Asylum, Wakefield.** The patients in the asylum at the end of 1915 numbered 2,562, as against 2,114 twelve months previously. Several important structural improvements were carried out during the year, including the erection and completion of a new chronic block.

Rates of Subscription: Twelve months, 6/6; Six months, 4/-; Three months, 2/-; post free to any address in the United Kingdom. Abroad, Twelve months, 8/8; Six months, 5/-; Three months, 2/6, post free. The Hospital "Index" to Volume LX., April to September 1916, is now ready, price 2½d. per copy, post free. Address The Manager, "The Hospital," The Hospital Building, 28/29 Southampton Street, Strand, London, W.C.

